# *In silico* Analysis of HIV-1 Env-gp120 Reveals Structural Bases for Viral Adaptation in Growth-Restrictive Cells

**DOI:** 10.3389/fmicb.2016.00110

**Published:** 2016-02-09

**Authors:** Masaru Yokoyama, Masako Nomaguchi, Naoya Doi, Tadahito Kanda, Akio Adachi, Hironori Sato

**Affiliations:** ^1^Laboratory of Viral Genomics, Pathogen Genomics Center, National Institute of Infectious DiseasesTokyo, Japan; ^2^Department of Microbiology, Institute of Biomedical Sciences, Tokushima University Graduate SchoolTokushima, Japan; ^3^Department of Research Promotion, Division of Infectious Disease Research, Japan Agency for Medical Research and DevelopmentTokyo, Japan

**Keywords:** homology modeling, MD simulation, V1/V2 loop, V3 loop, adaptive mutation

## Abstract

Variable V1/V2 and V3 loops on human immunodeficiency virus type 1 (HIV-1) envelope-gp120 core play key roles in modulating viral competence to recognize two infection receptors, CD4 and chemokine-receptors. However, molecular bases for the modulation largely remain unclear. To address these issues, we constructed structural models for a full-length gp120 in CD4-free and -bound states. The models showed topologies of gp120 surface loop that agree with those in reported structural data. Molecular dynamics simulation showed that in the unliganded state, V1/V2 loop settled into a thermodynamically stable arrangement near V3 loop for conformational masking of V3 tip, a potent neutralization epitope. In the CD4-bound state, however, V1/V2 loop was rearranged near the bound CD4 to support CD4 binding. In parallel, cell-based adaptation in the absence of anti-viral antibody pressures led to the identification of amino acid substitutions that individually enhance viral entry and growth efficiencies in association with reduced sensitivity to CCR5 antagonist TAK-779. Notably, all these substitutions were positioned on the receptors binding surfaces in V1/V2 or V3 loop. *In silico* structural studies predicted some physical changes of gp120 by substitutions with alterations in viral replication phenotypes. These data suggest that V1/V2 loop is critical for creating a gp120 structure that masks co-receptor binding site compatible with maintenance of viral infectivity, and for tuning a functional balance of gp120 between immune escape ability and infectivity to optimize HIV-1 replication fitness.

## Introduction

An envelope glycoprotein (Env) of human immunodeficiency virus type 1 (HIV-1) is synthesized in cells as a precursor gp160, and subsequently cleaved to mature gp120 and gp41. HIV-1 Env works, as a trimer of a gp120-gp41 dimer molecule, on viral entry into target host cells (Freed and Martin, 1995, 2013; Clapham and McKnight, 2002; Wilen et al., 2012). Env-gp120 binds to receptor CD4 and co-receptor CCR5 or CXCR4, whereas Env-gp41 mediates virus-cell membrane fusion. Depending on the co-receptor usage, HIV-1 strains are grouped into CCR5 (R5)-tropic and CXCR4 (X4)-tropic viruses. During the entry process, viral Env proteins undergo large conformational changes. CD4 induces gp120 conformational change upon their binding, and facilitates subsequent interaction with the co-receptor. Binding of gp120 to the receptor and co-receptor triggers a drastic structural change of gp41, and allows virus-cell membrane fusion. Successful entry is crucial for establishing HIV-1 infection.

HIV-1 Env displays high adaptive capacity. This ability enables HIV-1 to escape from host immune recognition with maintenance of function/structure of Env involved in viral entry. Consequently, HIV-1 Env exhibits an extensive genetic variation. Based on genetic variations, gp120 consists of discontinuous five conserved regions (C1 to C5) and five variable regions (V1 to V5; Freed and Martin, 1995). Numerous studies have detailed the role of each region in Env functions. V1/V2 and V3 form flexible loop structures on the gp120 core region (Huang et al., 2005; McLellan et al., 2011) and have been shown to be involved in modulation of the co-receptor usage, cell tropism, replication ability and/or neutralization resistance (Freed and Martin, 1995, 2013; Clapham and McKnight, 2002; Tilton and Doms, 2010; Benjelloun et al., 2012; Wilen et al., 2012; Connell and Lortat-Jacob, 2013; Mascola and Haynes, 2013). Recent crystal and cryo-electron microscopy (cryo-EM) structure studies on Env have revealed that V1/V2 loops are located at the trimer apex in connection with V3 loop, and that the conformational change of V1/V2 loops upon CD4 binding triggers exposure of V3 loop to the co-receptor (Julien et al., 2013; Lyumkis et al., 2013). These results are the indicative of concerted action of V1/V2 and V3 loops during HIV-1 entry and thereby adaptation processes. In this regard, we previously identified adaptive mutations in HIV-1 Env-gp120 that are responsible for viral growth enhancement in growth-restrictive cells of an R5-tropic and macaque/human tropic HIV-1 derivative, NL-DT562 (562; Nomaguchi et al., 2008, 2013a; Yamashita et al., 2008). These single-amino acid substitutions were found to be clustered within V1/V2 and V3 domains (Nomaguchi et al., 2013a).

Critical issues that remain to be solved for better understanding o f HIV-1 entry and adaptation can be summarized as follows: (1) amino acid residue(s) essential for the Env function are less well-defined; (2) adaptive mutation(s) that augment viral entry efficiency/replication ability are not yet amply described; (3) how the adaptive mutations alter viral phenotypes remain unclear. Although atomic-level information on the natural gp120 structure is essential to clarify each of these issues, such information is still technically hard to obtain. While the small-angle x-ray scattering (SAXS; Guttman et al., 2012) and cryo-EM (Lyumkis et al., 2013) have provided invaluable information on the global fold and shape of gp120 trimer on the virion, these techniques are in principle unable to give the atomic-level information on the protein structure. Meanwhile, the crystal structure analysis is beneficial to obtain the atomic-level information, but most studies have focused on the gp120 core region and are lacking in the flexible loop regions. To date, only a few studies have reported crystal structures of the full-length HIV-1 gp120 trimer (Julien et al., 2013; Pancera et al., 2014; Do Kwon et al., 2015). In those studies, protein preparations for crystallization were bound to anti-gp120 neutralizing antibodies, and/or contained multiple mutations at V3 base/C5 regions for structure stabilization and/or antigenicity modification. These physical modifications might influence structure and function of native gp120. Thus, information on the full-length gp120 structures obtained by distinct approaches would be informative to complement available structural data of the HIV-1 gp120.

In this study, we combined computational and experimental approaches to gain new insights into the roles of gp120 variable loops in HIV-1 entry and their adaptive mutations. By coupling homology modeling and molecular dynamics (MD) simulation, we constructed R5-tropic full-length gp120 models that disclose key structural features to understand gp120 functions: topologies of the variable loops (Guttman et al., 2012; Julien et al., 2013; Lyumkis et al., 2013; Pancera et al., 2014; Do Kwon et al., 2015), conformational masking of V3 tip (Guttman et al., 2012; Munro et al., 2014; Pancera et al., 2014), and CD4-induced conformational rearrangement (Huang et al., 2005; Guttman et al., 2012; Julien et al., 2013; Pancera et al., 2014; Do Kwon et al., 2015). Using the models and MD simulation, we obtained evidence that V1/V2 loop is critical to create gp120 structure that makes the masking of co-receptor binding site for immune escape compatible with the maintenance of viral infectivity. In parallel, cell-based viral adaptation experiments in the absence of antibodies led to the identification of single amino acid substitutions in Env gp120 for better viral entry and replication. Structural models predicted that these mutations resided in the receptors binding surface and could modulate gp120 structures for receptors binding. Our structural and virological data illustrate a previously less characterized physical function of V1/V2, i.e., the regulation of V3 conformation, for tuning viral infectivity and immune escape ability to optimize viral replication fitness in given environments.

## Materials and Methods

### Molecular Modeling of a Full-Length gp120 Monomer in CD4-Free and -Bound States

Three-dimensional (3-D) models for a full-length gp120 monomer of HIV-1 R5-tropic virus in CD4-free and -bound states were constructed by assembling missing parts of the protein surface onto the gp120 core of JRFL virus (GenBank accession no. U63632), followed by homology modeling. Comparison of gp120 core structures in public database indicated that overall conformations of the cores were similar in the CD4-free and -bound states except for V1/V2 stem region. Therefore, we used the core structure with gp41-interactive region of HIV-1 JRFL at a 2.61 Å resolution (PDB code:3JWD; Pancera et al., 2010) as a common platform to construct full-length gp120 models. Because 3JWD represents the core bound to soluble CD4, we replaced its V1/V2 stem region with that of the CD4-free partial core structure (PDB code: 3IDX, a 2.5 Å resolution; Chen et al., 2009) for modeling of gp120 in the CD4-free state. Other parts of gp120 were same for the present modeling. To supplement missing surface structures of gp120, we used partial structures as follows: V1/V2 (PDB code: 3U4E, a 2.19 Å resolution; McLellan et al., 2011), V3 (PDB code: 2QAD, a 3.3 Å resolution; Huang et al., 2007), V4 (PDB code: 2B4C, a 3.3 Å resolution; Huang et al., 2005), and C5 (PDB code: 1MEQ, solution nuclear magnetic resonance structure; Guilhaudis et al., 2002). The gp120 core and surface structures were used as templates to construct full-length models for JRFL gp120 by homology modeling using ‘MOE-Align,’ a tool for multiple sequence and structure alignment for examining their relatedness^1^, and ‘MOE-Homology,’ a tool to predict structure by the sequence and structure relatedness in the Molecular Operating Environment MOE (Chemical Computing Group Inc.). Obtained models were optimized by energy minimization using MOE and an Amber10: Extended Huckel Theory (EHT) force field implemented in MOE, which combines Amber10 and EHT bonded parameters for the large-scale energy minimization (Gerber and Muller, 1995; Case et al., 2005).

### MD Simulation of a Full-Length, Glycosylated gp120 in a CD4-Free State

A full-length, glycosylated gp120 model in a CD4-free state was subjected to MD simulation essentially as described for simulations of HIV-1/SIV gp120 outer domains (Naganawa et al., 2008; Yokoyama et al., 2012; Kuwata et al., 2013; Yuan et al., 2013). Briefly, a high-mannose oligosaccharide Man_5_GlcNAc_2_ was added to potential *N*-glycosylation sites in gp120 outer domains using Online Glycoprotein Builder^2^. MD simulations were performed by the PMEMD (Particle Mesh Ewald Molecular Dynamics) module in the AMBER 10 program package (Case et al., 2005), employing the AMBER ff99SB-ILDN force field, a protein force field with improved side-chain torsion potentials (Lindorff-Larsen et al., 2010), the GLYCAM06 force field, a biomolecular force field for glycans (Kirschner et al., 2008), and the TIP3P water model for simulations of aqueous solutions (Jorgensen et al., 1983). Bond lengths involving hydrogen were constrained with SHAKE, a constraint algorithm to satisfy a Newtonian motion (Ryckaert et al., 1977), and the time step for all MD simulations was set to 2 fs. A non-bonded cutoff of 10 Å was used. After heating calculations for 20 ps until 310 K using the NVT ensemble for the constant volume, temperature, and numbers of particles in the system, simulations were executed using the NPT ensemble for the constant pressure, temperature, and numbers of particles in the system at 1 atm, at 310 K, and in 150 mM NaCl for 50 ns.

### Molecular Modeling of HIV-1 gp120 Trimers in CD4-Free and -Bound States

For modeling of CD4-free trimer, the CD4-free monomer model was superposed on the gp120 trimer structure derived from the x-ray crystal structure of Env proteins (PDB code: 4TVP; Pancera et al., 2014). For modeling of CD4-bound trimer, the CD4-bound monomer model was superposed on the gp120 trimer structural image derived from cryo-EM analysis of Env proteins on the virion (PDB code: 3DNO; Liu et al., 2008). Superimpositions of monomer structures were done using the Protein Superpose module in MOE.

### Prediction of the Effects of Amino Acid Substitutions on the Stability and Affinity of gp120-CD4 Complex

The 3-D model of HIV-1 562 full-length gp120 bound to cynomolgus monkey CD4 (GenBank accession no. D63349) was constructed by homology modeling using MOE-Align and MOE-Homology, and refined by energy minimization and by the Ramachandran plot using MOE. The full-length gp120 model in a human soluble-CD4 bound state was used as a template for homology modeling. The 562 gp120 and cynomolgus monkey CD4 complex was used for *in silico* mutagenesis as described in the study of HIV-1 capsid protein (Nomaguchi et al., 2013b). Changes in the stability and affinity of the gp120-CD4 complex by mutations were computed by using the Protein Design application in MOE, a computational tool for the structure analysis of mutant proteins and for the computational design of protein with desirable properties. Briefly, single-point mutations on the gp120 protein were generated, and ensembles of protein conformations were generated by the LowMode MD module in MOE, a tool of low-mode velocity filtering for conformational search, to calculate average stability and affinity using Boltzmann distribution. Finally, stability and affinity scores of the structures refined by energy minimization were obtained through the scoring function of the Protein Design application.

### MD Simulation of gp120 V3 Loops

Molecular models for V3 loops of 562 and 562 S304G gp120 proteins were constructed by homology modeling using the x-ray crystal structure of gp120 with V3 loop at a 3.30 Å resolution (PDB code: 2QAD; Huang et al., 2007) as a modeling template. The models represent V3 loop structures of gp120 in the CD4-bound state, because the template gp120 was bound to soluble CD4. The initial V3 models were thermodynamically and physicochemically refined using MOE. MD simulations of V3 loops were done essentially with the same calculation conditions as described above for MD simulations of the full-length gp120. After heating calculations for 20 ps until 310 K using the NVT ensemble, simulations were executed using the NPT ensemble at 1 atm and at 310 K for 20 ns. Superimpositions of V3 structures were done using the Protein Superpose module in MOE by coordinating atoms of amino acid residues at V3 base.

### Calculation of Root Mean Square Deviation (RMSD) and Root Mean Square Fluctuation (RMSF) Values

Root Mean Square Deviation and RMSF values were calculated as previously described to quantify structural dynamics of molecules in the MD simulations (Yokoyama et al., 2012). RMSD values between the heavy atoms of the two superposed proteins were used to measure overall structural differences between the two proteins (Case et al., 2005). We also calculated RMSF values of the Cα atoms to obtain information on atomic fluctuations of individual amino acid residues during MD simulations (Case et al., 2005). The 10,000 snapshots obtained from MD simulations of 10–20 ns were used to calculate RMSF values. The average structures during the last 10 ns of MD simulations were used as reference structures for RMSF calculation. Both RMSD and RMSF values, which quantify the differences between the average values and those obtained at give times of MD simulations, were calculated using the ptraj module in Amber, a trajectory analysis tool (Case et al., 2005).

### Plasmid DNA

Construction and characterization of various proviral clones designated pNL-DT562, pNL-DT5R, pNL4-3, and pNL-Kp (NL4-3 ΔEnv construct) were previously described (Adachi et al., 1986, 1991; Kamada et al., 2006; Yamashita et al., 2008). Growth-enhancing mutations were site-specifically introduced into pNL-DT562 by using the QuickChange site-directed mutagenesis kit (Agilent Technologies) as fully described previously (Nomaguchi et al., 2013a, 2014).

### Cells

A human monolayer cell line 293T (Lebkowski et al., 1985) and a cynomolgus macaque lymphocyte cell line HSC-F (Akari et al., 1999) were routinely maintained in Eagle’s MEM supplemented with 10% heat-inactivated fetal bovine serum and in RPMI1640 containing 10% heat-inactivated fetal bovine serum, respectively.

### Transfection, Reverse Transcriptase (RT) Assay, and Infection

Virus samples were prepared from 293T cells transfected with various proviral clones by the calcium-phosphate co-precipitation method as previously described (Adachi et al., 1986; Kamada et al., 2006; Nomaguchi et al., 2013a,b, 2014). Virion-associated RT activity was measured to determine virus amounts as described previously (Willey et al., 1988; Nomaguchi et al., 2013b). For determination of viral growth kinetics, equal amounts (RT units) of virus preparations were inoculated into HSC-F cells, and infected cells were cultured in the presence of IL-2 (50 U/ml). Virus replication was monitored every 3 days by RT activity in the culture supernatants.

### Entry Assay

Input virus samples for entry assays were prepared from transfected 293T cells as above, and quantified by the HIV-1 p24 antigen enzyme-linked immunosorbent assay (ELISA) kit (ZeptoMetrix Corporation). Entry assays were performed as previously described (von Schwedler et al., 1993; Adachi et al., 1996). Briefly, HSC-F cells (10^6^) were incubated with viruses (100 ng of p24) for 2 h at 4°C, extensively washed, and collected as viral binding fractions. To determine virus entry level, HSC-F cells treated with viruses at 4°C as above were trypsinized for 5 min at 37°C, extensively washed, and incubated for 2 h at 37°C for preparation of viral entry fractions. The binding and entry fractions were lysed, and the p24 level of samples was determined by the ELISA kit as above. Entry efficiency of each sample was calculated as p24 level of entry fraction/p24 level of binding fraction. NL4-3 and NL-Kp (NL4-3ΔEnv) were used as positive and negative controls, respectively.

### Inhibitor Sensitivity Assays

To determine the co-receptor usage, HSC-F cells (10^5^) were treated with/without 1 μM co-receptor antagonists (CXCR4 antagonist AMD3100 and CCR5 antagonist TAK-779) at 37°C for 1 h, and equal virus amounts (10^5^ RT units) were inoculated into the pretreated cells. Co-receptor antagonists were present in the cultures throughout the experiment. Virus replication was monitored by RT activity released into the culture supernatants. Viral yields in test cultures relative to those on the peak day in cultures without antagonists were calculated. X4-tropic HIV-1 derivative 5R served as a control. To determine sensitivity of 562 and its gp120-mutants to TAK-779, HSC-F cells (10^5^) were treated with serial 10-fold dilutions of TAK-779 (1–10^-5^ μM) at 37°C for 1 h, and viruses (10^6^ RT units for 562 and 10^5^ RT units for gp120-mutants) were then inoculated into the pretreated cells. TAK-779 concentration was maintained in the cultures throughout the experiment. Virus replication was monitored by RT activity released into the culture supernatants. Viral yields in test cultures relative to those on the peak day in cultures without TAK-779 were calculated.

## Results

### MD Simulation of a Full-Length, Glycosylated gp120 Protein Predicts a Thermodynamically Stable Arrangement of V1/V2 and V3 Loops in the CD4-Free State

**FIGURE 1:**
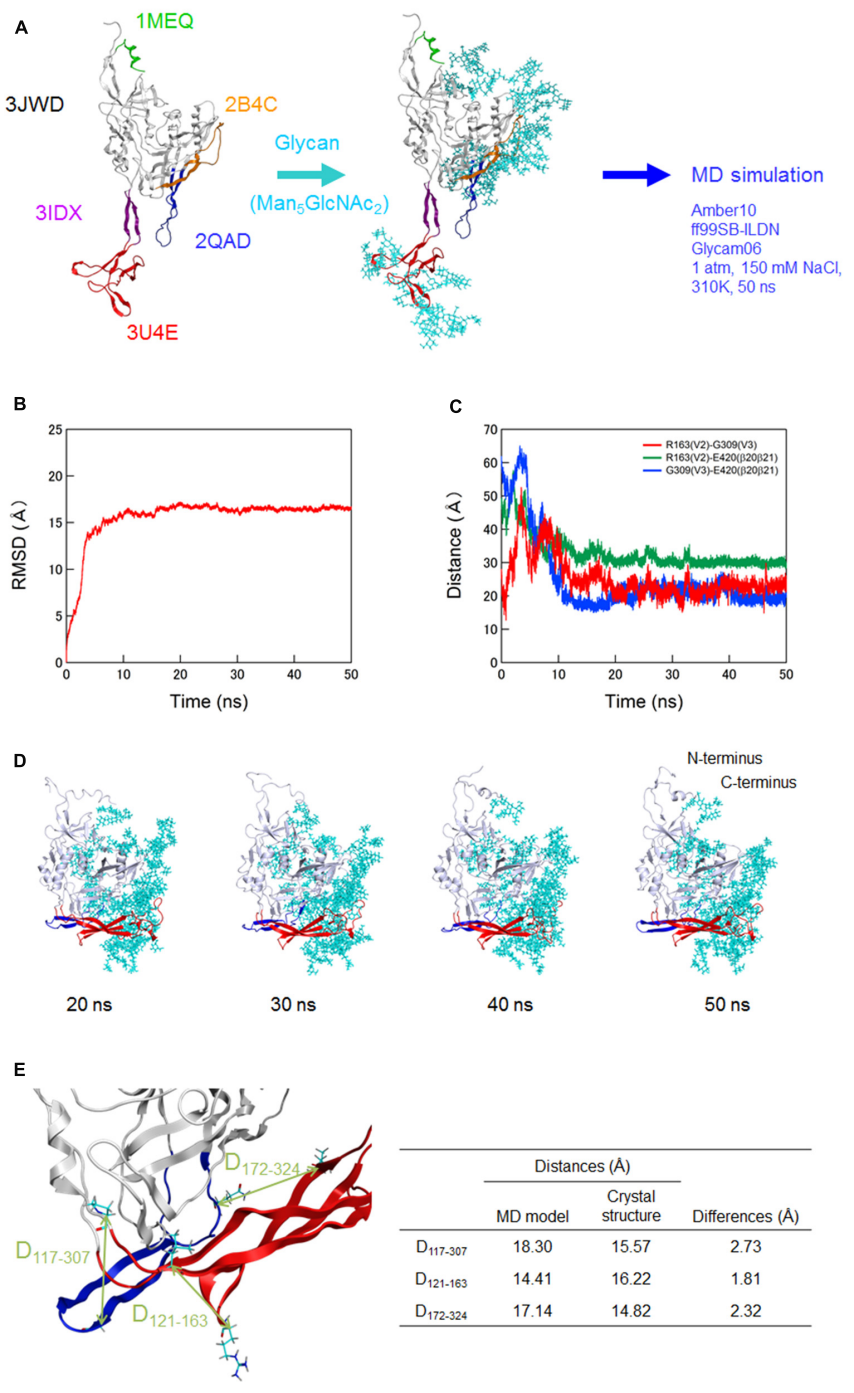
**Molecular dynamics (MD) simulation of a full-length, glycosylated HIV-1_JRFL_ gp120 monomer. (A)** Schematic representation of molecular modeling and MD simulation. Molecular model for a full-length, glycosylated gp120 monomer of HIV-1 R5-tropic virus JRFL in a CD4-free state was constructed by assembling of HIV-1 gp120 parts and homology modeling. PDB codes of the structures used for modeling are indicated on the left. Thermodynamically and physically refined model was subjected to MD simulation using the PMEMD module in the AMBER 10 program package as described for simulations of HIV-1/SIV gp120 outer domains (Naganawa et al., 2008; Yokoyama et al., 2012; Kuwata et al., 2013; Yuan et al., 2013). **(B)** Time course of RMSD between the initial model and models at given times of MD simulation. RMSD values were calculated with trajectories at every 2 fs of MD simulation using the ptraj module in Amber 10. **(C)** Time course of distances between residues in V2 and V3 (red line), residues in V2 and β20-β21 (green line), and residues in V3 and β20-β21 (blue line) in gp120. **(D)** Structures of a full-length, glycosylated gp120 monomer at 20, 30, 40, and 50 ns of MD simulations. V1/V2 and V3 regions are highlighted by red and blue colors, respectively. **(E)** Distances between the variable loops and core. The distances between the Cα of P117 at core neighboring V1 base and Cα of G307 at the tip of V3 loop (D_117-307_), Cα of L121 at core neighboring V1 base and the Cα of R163 at V2 loop (D_121-163_), and Cα of L172 at V2 loop and the Cα of Q324 at V3 base (D_172-324_) were calculated with the gp120 model at 50 ns simulation and x-ray crystal structure (4TVP) to quantitatively compare relative 3-D locations of the V1/V2 and V3 loops on the core. Amino acid numbers are based on those of the JRFL Env protein (GenBank accession no. U63632).

Because full-length gp120 structures of the same strain in CD4-free and -bound states were unavailable at atomic levels in the public database, we constructed them with the aid of *in silico* science (see Materials and Methods for details of modeling). Unliganded gp120 protein of HIV-1 is characterized to exist as semi-stable state, i.e., local minimum of energy state, through interactions of variable loops on the core surface (Kwon et al., 2012). Maintaining such unique conformation is considered to be critical for generating its functions in virus entry into target cells and in immune/drug escape (Kwon et al., 2012). To identify the functional arrangement of the variable loops at semi-stable state of unliganded gp120 protein, we performed MD simulation for the CD4-free gp120 model (**Figure 1A**). To mimic physical properties of the native gp120, we added high-mannose oligosaccharide Man_5_GlcNAc_2_ at the potential N-glycosylation sites in gp120 outer domains of the initial model before MD simulation. RMSD between the initial structure and structures at given times of simulation sharply increased in the beginning and reached a near plateau after 20 ns of the MD simulations (**Figure 1B**). These results suggest that the structural distortions were relieved shortly after the start of simulation under thermodynamic driving forces in solution. The data also predict that the glycosylated gp120 structure can reach a state of thermodynamic equilibrium in solution.

Of note, distances between V2 and V3, V2 and β20–β21, and V3 and β20–β21 in gp120 reached a near plateau with low levels of fluctuations (**Figure 1C**). The data indicate that a stable arrangement of V1/V2 and V3 on the glycosylated gp120 core exists in solution in the CD4-free state. The data also suggest that V1/V2 and V3 interactions confine their free movements. The results are consistent with a previous report on the physical function of V1/V2 loop for the regulation of gp120 conformation (Kwon et al., 2012). In this MD-predicted equilibrium state, V1/V2 and V3 loops positioned at the opposite side from the N- and C-terminal regions and continually fluctuated over time (**Figures 1C,D**). V1/V2 was extended from inner to outer domains along core, whereas V3 was extended from outer to inner domains along core (**Figure 1D**). The relative 3-D locations of V1/V2 loop, V3 loop, and N/C termini on the core in the equilibrium state agree with those reported in the structural model constructed with SAXS data of a CD4-free full-length gp120 (Guttman et al., 2012). The relative 3-D locations also agree with those in the cryo-electron tomography reconstruction model (Lyumkis et al., 2013) and the x-ray crystal structures (Julien et al., 2013; Pancera et al., 2014; Do Kwon et al., 2015) for gp140 containing whole gp120 and the ectodomain of gp41. To quantitatively address this issue, we measured the distances between the variable loops and core at three sites (D_117-307_, D_121-163_, and D_172-324_) using our gp120 model at 50 ns simulation and x-ray crystal structure at pre-fusion state (4TVP; **Figure 1E**). The differences in distances between the model and x-ray crystal structure were 2.73, 1.81, and 2.32 Å for the D_117-307_, D_121-163_, and D_172-324_, respectively, which were within the range of resolution of the 4TVP (3.1 Å). Thus, the gp120 structure model at 50 ns in the equilibrium state closely recapitulates relative 3-D locations of V1/V2 and V3 loops on the core of the x-ray crystal structure.

### Conformational Masking of a Co-Receptor Binding Site in the Ligand-Free, Trimeric gp120

**FIGURE 2:**
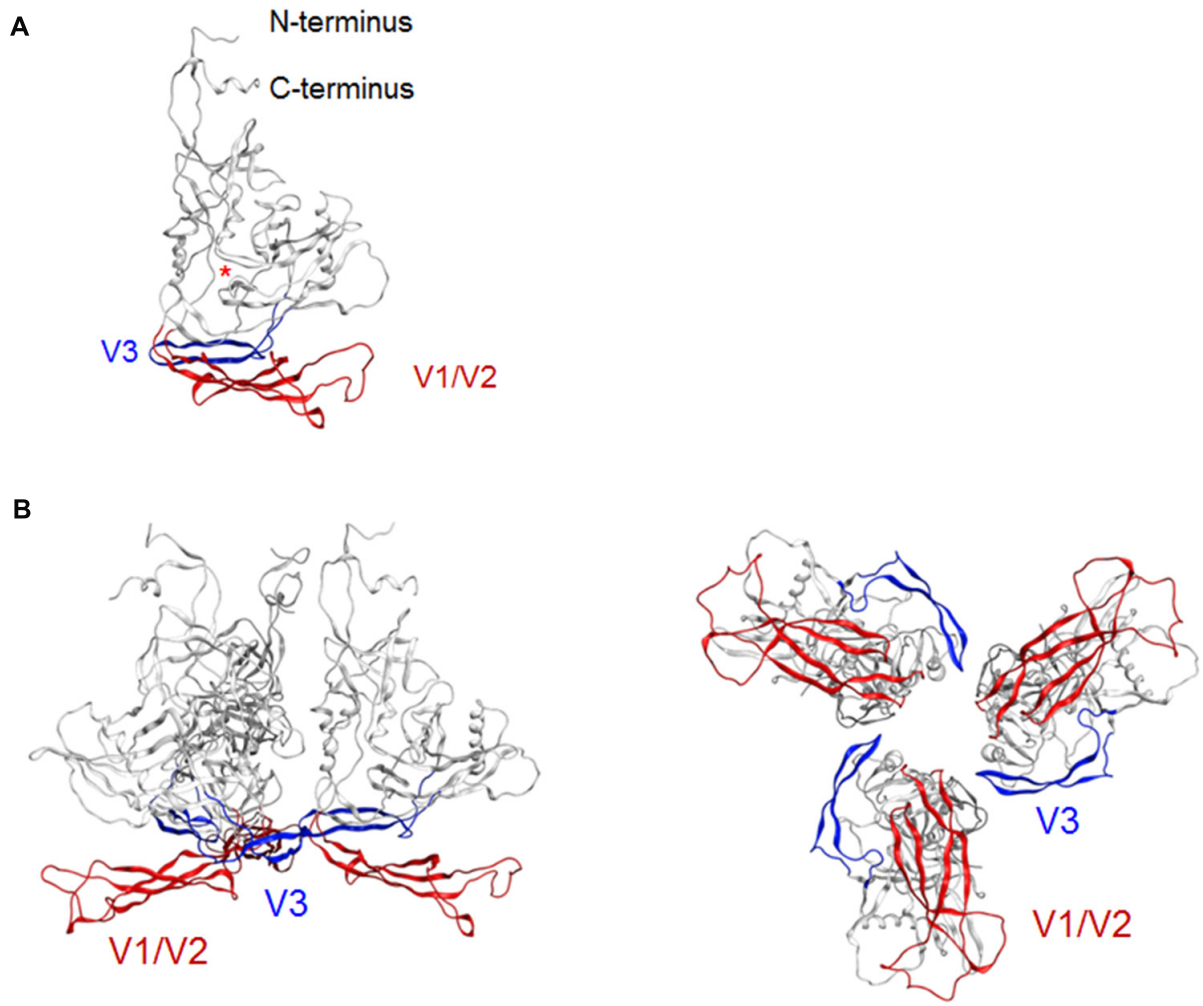
**Model for a full-length HIV-1_JRFL_ gp120 in a CD4-free state. (A)** Full-length gp120 monomer model. The structure at 50 ns of MD simulation in **Figure 1D** is shown. Glycans are removed for clear view of the outer domain. A red asterisk indicates the location of CD4-binding loop. **(B)** Full-length gp120 trimer model. The model was constructed by superposing CD4-free monomer model on the x-ray crystal structure of Env gp140 protein (PDB code: 4TVP; Pancera et al., 2014). Side and top views are shown on the left and on the right, respectively. V1/V2 and V3 regions are highlighted by red and blue colors, respectively.

Env gp120 V3 loop element is critical for co-receptor binding for HIV-1 entry into target cells and determines viral co-receptor tropism (Hwang et al., 1991). The tip region of V3 loop therefore constitutes a potent neutralization epitope (Goudsmit et al., 1988; Palker et al., 1988; Rusche et al., 1988; Javaherian et al., 1989). However, studies on viral neutralization (Bou-Habib et al., 1994; Stamatatos and Cheng-Mayer, 1995; Krachmarov et al., 2005; Lusso et al., 2005), gp120 conformational dynamics (Munro et al., 2014), and V3 sequence evolution (Sato et al., 1999; Shiino et al., 2000) consistently indicate that this epitope is generally concealed from the antibody access in the primary isolates. HIV-1 R5-tropic virus clone JRFL used in the present modeling study is also a neutralization-resistant tier-2 primary isolate from an HIV-1 infected individual (Koyanagi et al., 1987). To assess whether our model here predicts this conformational masking on virion, we constructed a full-length gp120 trimer model by superposing the structure at 50 ns of MD simulation on the x-ray crystal structure of envelope gp140 trimer (PDB code: 4TVP; **Figure 2**). Whereas V3 tip was exposed on the gp120 at the monomeric state (**Figure 2A**), this region was positioned toward the center at the trimeric state so that V3 tip is sterically concealed (**Figure 2B**). These results are consistent with those in the SAXS-based gp120 model (Guttman et al., 2012), the x-ray crystal structure of gp140 (Pancera et al., 2014), and the single-molecule fluorescence resonance energy transfer imaging (Munro et al., 2014).

### Conformational Rearrangement of V1/V2 and V3 Loops After CD4 Binding

**FIGURE 3:**
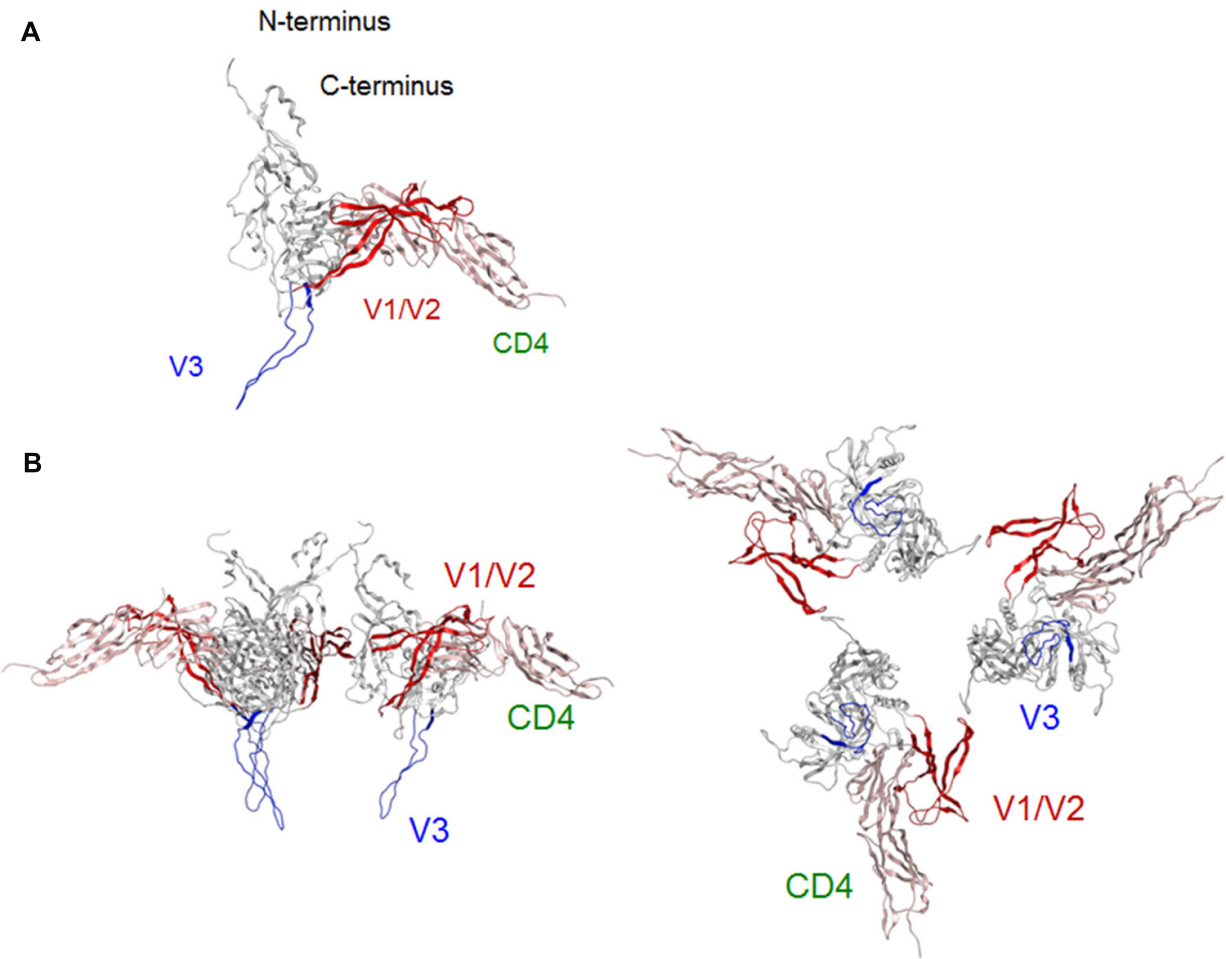
**Model for a full-length HIV-1_JRFL_ gp120 in a CD4-bound state. (A)** Full-length gp120 monomer model. Molecular model for a soluble CD4-bound full-length gp120 monomer was constructed as described in section “Materials and Methods.” **(B)** Full-length gp120 trimer model. The model was constructed by superposing CD4-bound monomer model on the Env structure derived from cryo-EM analysis (PDB code: 3DNO; Liu et al., 2008). Side and top views are shown on the left and on the right, respectively. V1/V2 and V3 regions are highlighted by red and blue colors, respectively.

Virological (Moore et al., 1992; Lee et al., 1997) and structural (Huang et al., 2005; Julien et al., 2013; Lyumkis et al., 2013; Pancera et al., 2014; Do Kwon et al., 2015) studies consistently suggest that following CD4 binding, the gp120 surface undergoes conformational changes from co-receptor-binding-incompetent to -competent form. To address this issue, we constructed a full-length gp120 model in a CD4-bound state (**Figure 3A**). The global shape and structural topologies of variable loops of this model are consistent with those in the SAXS-based gp120 model in the CD4-bound state (Guttman et al., 2012). Comparison of our gp120 models in the CD4-free (**Figure 2A**) and -bound (**Figure 3A**) states shows that the core structures of the two models are similar except for the secondary structure of V1/V2 stem. This difference, however, caused marked difference in arrangements of V1/V2 and V3 loops on the core. In the CD4-free state, V1/V2 was positioned near V3 loop so that the variable surfaces could interact with each other to maintain a stable arrangement on the core (**Figures 1B–D**, and **2A**). In the CD4-bound state, however, V1/V2 loop is rearranged near the bound CD4 to support CD4 binding: V1/V2 stem formed bridging sheet in combination with β20–β21 loop so that V1/V2 loop could interact with CD4 instead of V3 (**Figure 3A**). This V1/V2 repositioning is consistent with the SAXS-based gp120 model for the CD4-bound state (Guttman et al., 2012). Due to this conformational rearrangement in the CD4-bound state, V3 loop became free from structural constraints imposed by the V1/V2 interactions and protruded out of the core surface for co-receptor interactions in the trimer state (**Figure 3B**). These results agree with what was proposed previously for the conformational rearrangements of gp120 variable surface upon CD4 binding (Guttman et al., 2012).

### Each Individual Adaptive Single-Amino Acid Mutation in V1/V2 and V3 Regions of 562 Env-gp120 Enhances Viral Growth Potential

Our structural study suggests that V1/V2 region is critical for determining viral phenotypes via regulating topology and conformation of V3 loop. Considering that V1/V2 and V3 loop are variable, we assumed that these regions might be critical for adaptive mutations under changing environments.

To address this issue, we examined here how HIV-1 adapts in our cell-based HIV-1 assay system (Kamada et al., 2006; Nomaguchi et al., 2008, 2013a). While multiple selective factors including the host immune response drive virus adaptation/evolution in individuals, only those that affect viral replication ability itself contribute to virus adaptation in the cell culture system, providing simple experimental means to study HIV-1 adaptation for entry. Previously, we performed continuous cultivation of 562 and its X4-tropic version NL-DT5R (5R) under growth-restrictive conditions imposed by macaque cell lines (Nomaguchi et al., 2013a). In our repeated experiments, 562 and 5R frequently acquired growth-enhancing adaptive mutations in both Pol-integrase and Env-gp120 regions (Nomaguchi et al., 2013a, 2014).

**FIGURE 4:**
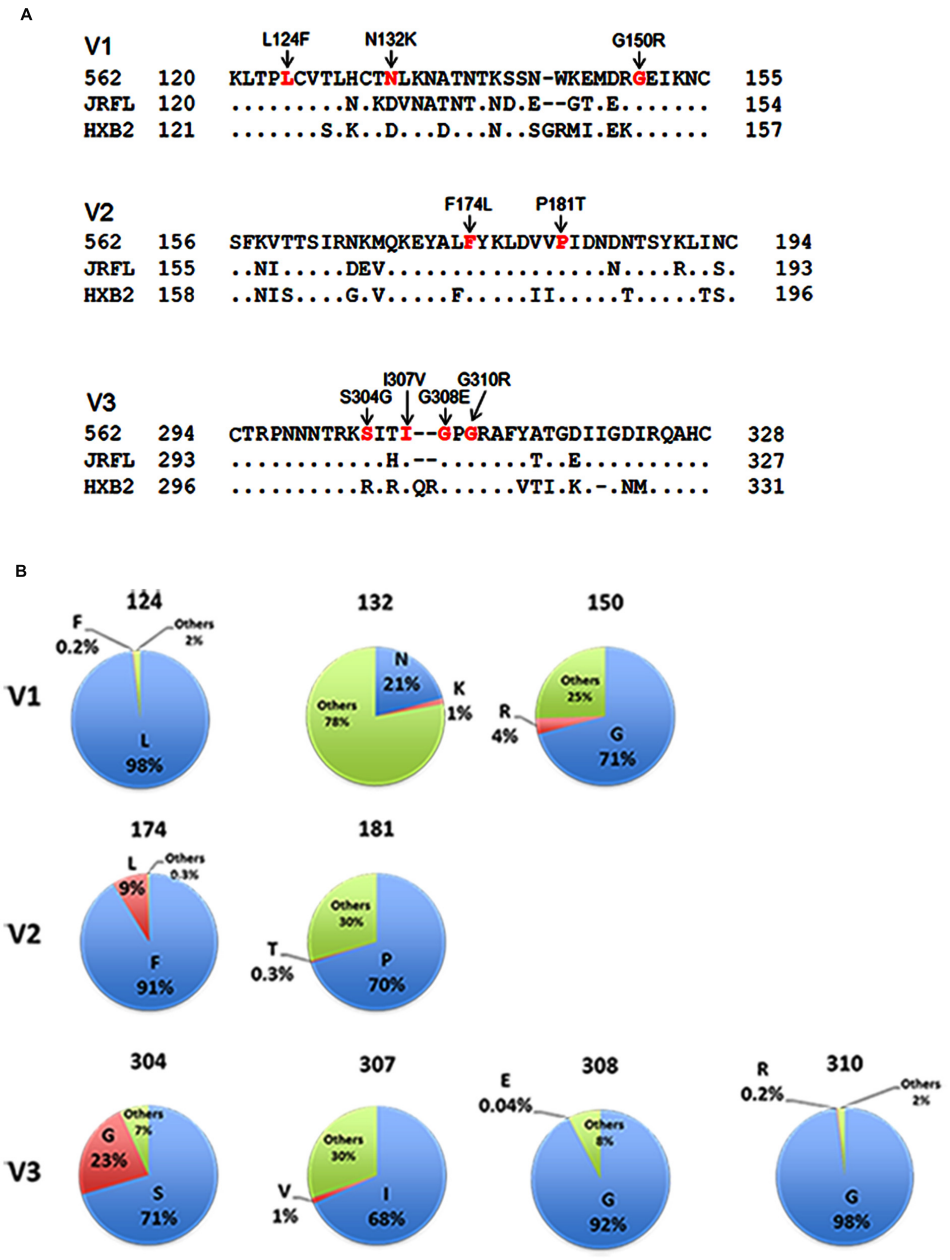
**Genetic information on HIV-1 gp120 adaptive mutations in the present study. (A)** Location of HIV-1 adaptive mutations in Env-gp120. Amino acid sequences in V1 to V3 regions of HIV-1 R5-tropic 562 virus (Nomaguchi et al., 2013a) carrying the SF162 *env* gene (GenBank accession no. EU123924; Kawamura et al., 1994) are aligned with those of R5-tropic JRFL clone (GenBank accession no. U63632) and of X4-tropic HXB2 clone (GenBank accession no. K03455). Assignment of V1, V2, and V3 regions is based on gp120 structure: V1/V2 (PDB code: 3U4E), V3 (PDB code: 2QAD). **(B)** Frequency of authentic (light blue) and replaced (red) amino acid residues. Others (green) represent amino acid residues other than authentic and replaced ones. Naturally occurring amino acid residues at specific positions, where adaptive 562-gp120 mutations are located, were investigated in an HIV-1 subtype B population from different geographic regions in the world (19,419 sequences), and are graphically shown. The 19,419 sequences were obtained from the HIV Sequence Database (http://www.hiv.lanl.gov/content/sequence/HIV/mainpage.html).

**FIGURE 5:**
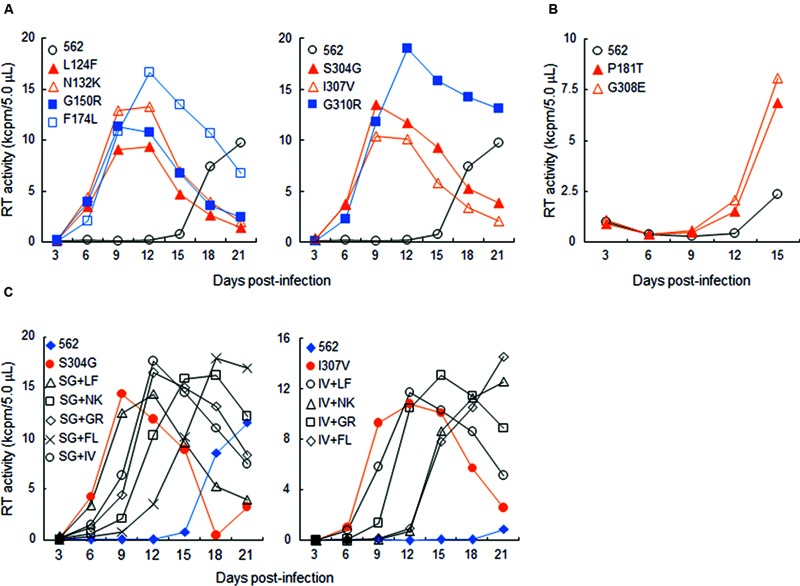
**Growth properties of 562 derivative viruses carrying various adaptive mutations in gp120. (A)** Growth kinetics of variant viruses carrying a single adaptive mutation. Virus samples were prepared from 293T cells transfected with indicated proviral clones, and inoculated into HSC-F cells. Virus replication was monitored by RT activity released into the culture supernatants. Data of left and right panels were obtained from the same experiment, and the same result for 562 is separately shown as a control. Infection condition: 2 × 10^5^ RT units/2 × 10^5^ cells. **(B)** Growth properties of slowly growing variant viruses carrying a single adaptive mutation. Experiment was performed as described above. Infection condition: 4 × 10^6^ RT units/10^6^ cells. **(C)** Growth kinetics of variant viruses carrying two adaptive mutations. Experiments were performed as above. LF, L124F; NK, N132K; GR, G150R; FL, F174L; SG, S304G; IV, I307V; GR, G310R. Infection conditions: left, 2 × 10^5^ RT units/2 × 10^5^ cells; right, 5 × 10^4^ RT units/1 × 10^5^ cells.

In this study, we focused on analyzing the changes in the 562 Env-gp120 to understand structural bases for growth adaptation. Remarkably, the nine single-amino acid substitutions frequently emerged during the adaptation experiments were all positioned at V1/V2 or V3 domain (Nomaguchi et al., 2013a; **Figure 4A**). Authentic amino acid residues at the mutation sites are relatively well-conserved among HIV-1 strains, and represented the most dominant ones (**Figure 4B**). Substituted amino acid residues by the adaptations were all found in nature, but usually at much lower frequencies as compared with the authentic ones (**Figure 4B**). These data suggest that the nine sites are intrinsically variable in nature, yet receive constraints on changes under standard replication and transmission conditions. To address biological impacts of these single substitutions, we comparatively analyzed the growth-potentials of a parental clone 562 and its mutants carrying each amino acid substitution. Seven substitutions, L124F, N132K, G150R, F174L, S304G, I307V, and G310R, markedly enhanced replication ability relative to that of 562 (**Figure 5A**). Furthermore, these mutations except for G310R similarly promoted viral growth in human cells (data not shown). The effect of P181T and G308E substitutions on viral growth was modest (**Figure 5B**), and therefore, these substitutions were not included in the subsequent analyses. To examine whether a combination of substitutions has an additive enhancing effect, we constructed proviral double mutant clones containing various combinations of substitutions, and compared their replication abilities with those of parental 562 and single mutant viruses (**Figure 5C**). Although viruses carrying combined substitutions in V1/V2 and V3 grew more efficiently than 562, additive enhancing effects resulted from these double substitutions and a double V3 substitution (S304G and I307V) were not observed. Similar results were obtained for the other double substitutions of V1/V2 and V3 loops (data not shown).

### Adaptive Mutations in 562 Env-gp120 Contribute to Increase in Entry Efficiency via the Enhancement of Affinity for CCR5

**FIGURE 6:**
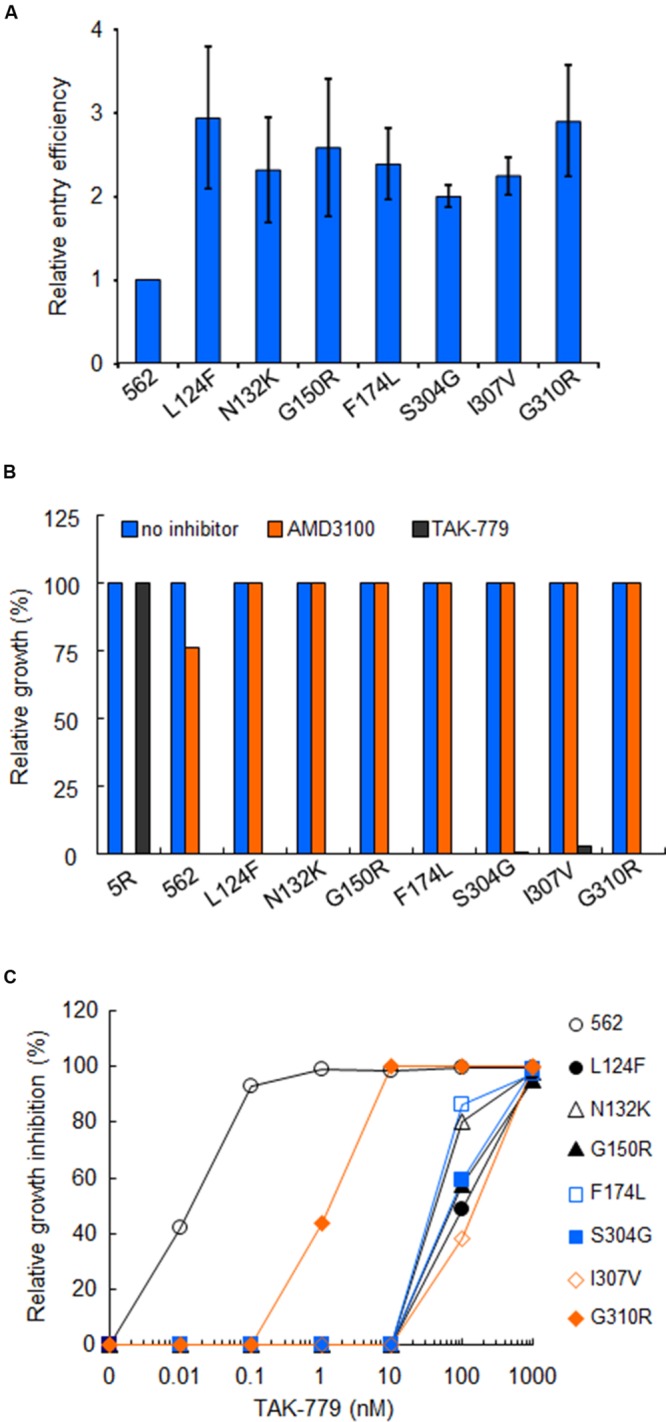
**Effects of growth-enhancing mutations in gp120 on viral entry. (A)** Entry efficiency into HSC-F cells of 562 and 562 carrying indicated single substitutions. Virus samples were prepared as in **Figure 5**, and entry assays were performed as described in section “Materials and Methods.” Values obtained for ΔEnv construct (NL-Kp) were subtracted from those for test samples. Entry efficiency of each virus relative to that of 562 is presented. **(B)** Co-receptor usage of various viruses. Infection of HSC-F cells with viruses was performed as described above, and infected cells were cultured in the absence or presence (1 μM) of antagonists (CXCR4 antagonist AMD3100 or CCR5 antagonist TAK-779). Virus replication was monitored by RT activity released into the culture supernatants. Viral yields in test cultures relative to those on the peak day in cultures without antagonists were determined. 5R and 562 served as controls. **(C)** Sensitivity of 562 and its mutants to TAK-779. Virus samples prepared as above were inoculated into HSC-F cells pretreated with the indicated concentration of TAK-779. Virus replication was monitored by RT activity released into the culture supernatants. Viral yields in test cultures relative to those on the peak day in cultures without TAK-779 were determined and presented as % inhibition. Representative results from three independent experiments are shown.

Next, we examined the effect of these substitutions on viral entry efficiency. Virus samples were prepared from 293T cells transfected with proviral constructs (562 and 562 mutants carrying each growth-enhancing substitution). Entry assays in HSC-F cells were then performed in a two-step reaction: virus binding step in which virus-cell mixtures were kept at 4°C; subsequent virus penetration step in which cells after virus binding were incubated at 37°C. Entry efficiency of each virus sample was calculated as p24 level of penetrated virus into cells relative to that of attached virus onto cells. Notably, all the seven substitutions enhanced the viral entry efficiency in cells (**Figure 6A**). These data suggest that adaptive mutations in 562 Env-gp120 promote viral replication by increasing entry efficiency.

Since it has been shown that alterations in V1/V2 and V3 regions modulate the co-receptor usage (Freed and Martin, 1995, 2013; Clapham and McKnight, 2002; Tilton and Doms, 2010; Benjelloun et al., 2012; Wilen et al., 2012; Connell and Lortat-Jacob, 2013; Mascola and Haynes, 2013), we determined the susceptibility of above mutants to co-receptor antagonists. Virus samples were prepared from 293T cells transfected with proviral constructs (5R, 562 and 562 mutants carrying each growth-enhancing substitution), and inoculated into HSC-F cells pretreated with the antagonists. As is clear in **Figure 6B**, the replication of 5R was inhibited in the presence of AMD3100 (anti-CXCR4), but not TAK-779 (anti-CCR5). Conversely, the replication of 562 and its single-amino acid mutants (L124F, N132K, G150R, F174L, S304G, I307V, and G310R) was restricted in the presence of TAK-779, but not AMD3100 (**Figure 6B**). These results indicate that the viral co-receptor tropism is not affected at all by the above mutations in Env-gp120.

It has been demonstrated that the Env with enhanced affinity for co-receptor displays increased resistance to entry inhibitors (e.g., T-20) and co-receptor antagonists (e.g., TAK-779; Reeves et al., 2002; Lobritz et al., 2007; Pacheco et al., 2008). Therefore, we compared the sensitivity of 562 and its mutants to TAK-779 (**Figure 6C**). HSC-F cells pretreated with increasing concentrations of TAK-779 were infected with 562 or its mutants (L124F, N132K, G150R, F174L, S304G, I307V, or G310R). A high concentration of TAK-779 was required to completely restrict the replication of viral clones containing mutations L124F/N132K/G150R/F174L/S304G/I307V, and a mutant clone carrying G310R was modestly more resistant to TAK-779 relative to 562. These results show that the growth-enhancing mutations in 562 Env-gp120 clearly enhance, without exception, the affinity for CCR5. Collectively, the results obtained show that adaptive mutations in 562 Env-gp120 promote viral replication by enhancing entry efficiency through increase in the affinity for CCR5.

### Three-D Locations and Structural Impacts of the Adaptive Mutations

**FIGURE 7:**
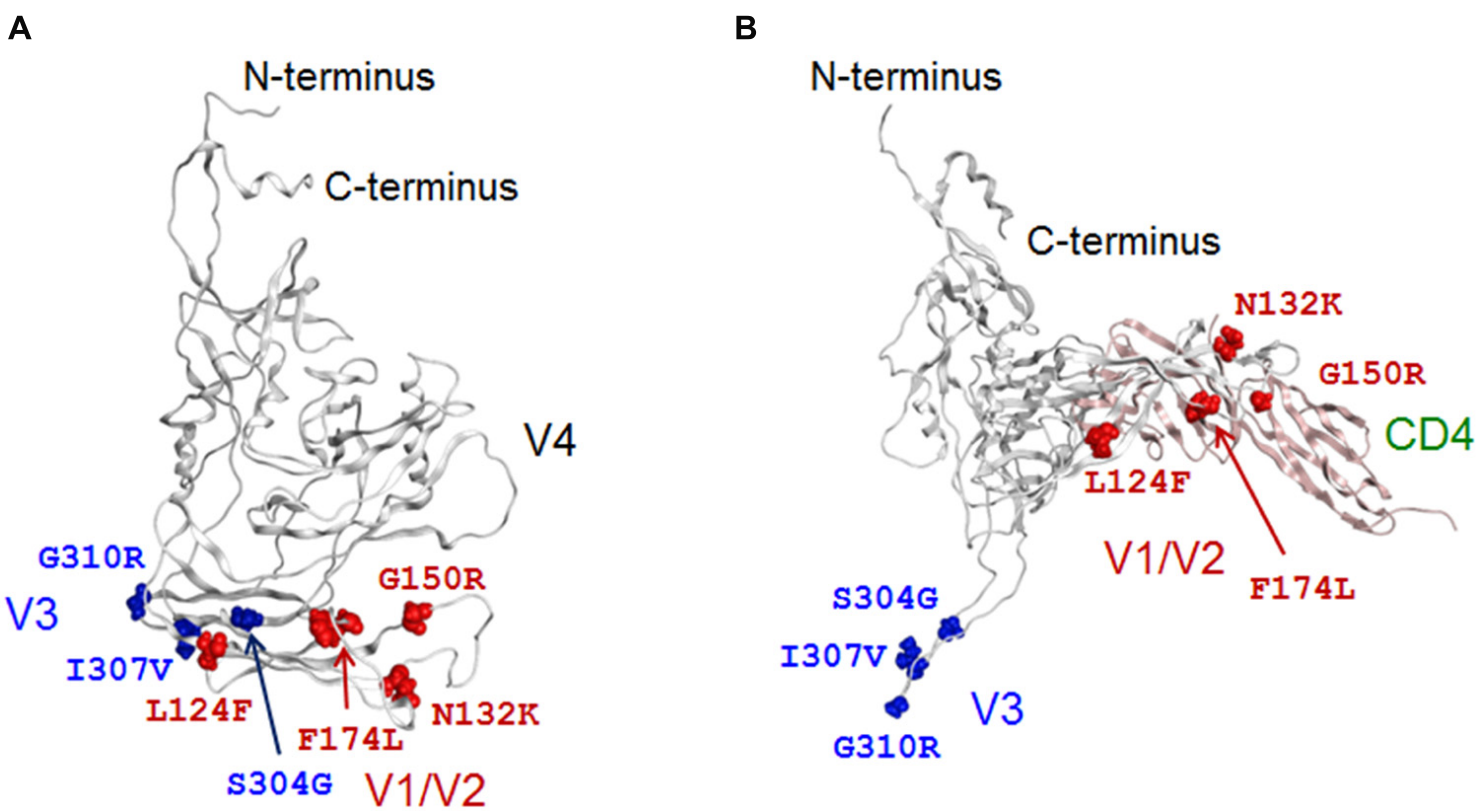
**The 3-D locations in a full-length gp120 of adaptive mutations.** Adaptive amino acid substitutions are highlighted by colored globules on the gp120 models in CD4-free **(A)** and CD4-bound **(B)** states. Amino acid residues in V1/V2 and V3 regions are highlighted by red and blue colors, respectively. For details of the two models, see **Figures 2A** and **3A**.

In parallel with the above experimental study, we investigated 3-D locations and possible structural impacts of the adaptive mutations to gain insights into how the mutations change the viral phenotype. Interestingly, whereas the seven substitutions were sporadically distributed along the gp120 core surface before CD4 binding (**Figure 7A**), all of them were rearranged at or near the potential sites for CD4 or co-receptor binding after CD4 binding (**Figure 7B**): they were positioned either around the CD4 binding surface in the V1/V2 region or at the V3 tip/stem region. The data suggest that some of these mutations may influence the gp120 structure for receptors binding.

**FIGURE 8:**
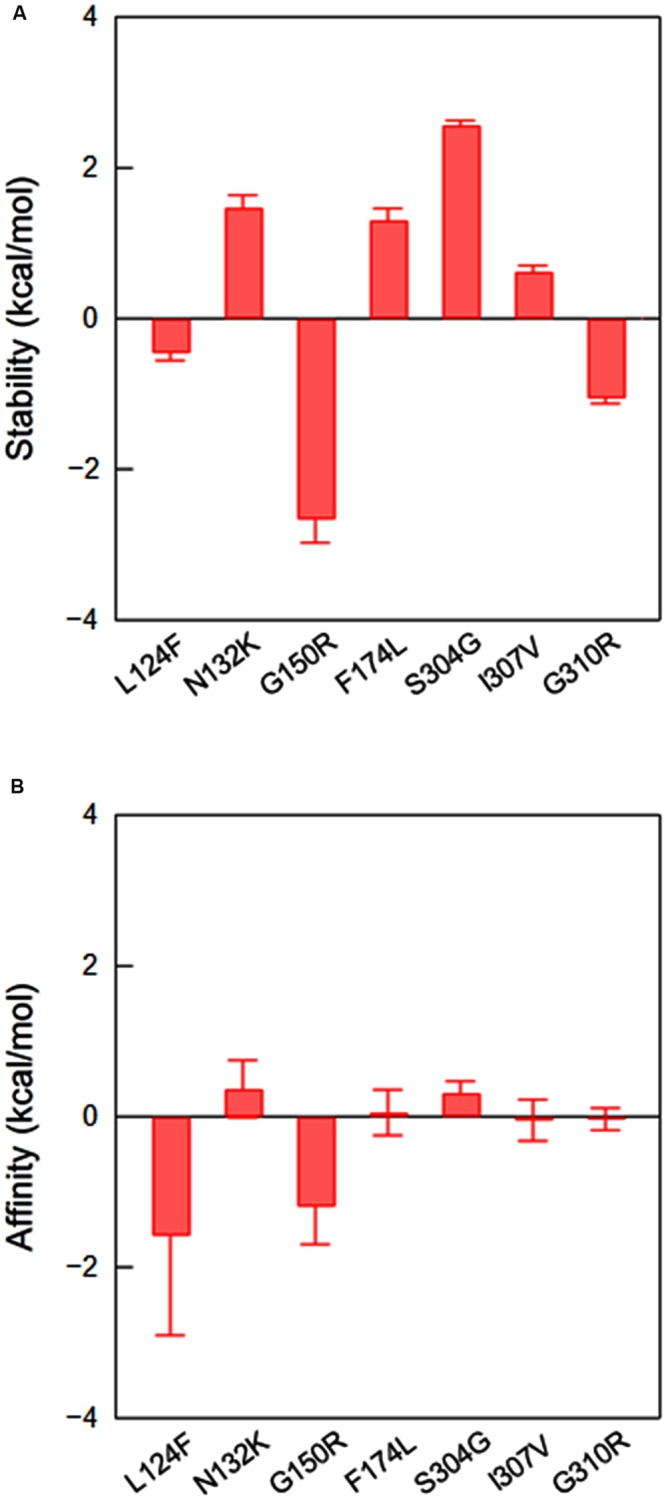
**Effects of amino acid substitutions on the stability and affinity of gp120-CD4 complex.** Full-length 562 gp120-CD4 complex model was constructed as described in section “Materials and Methods,” and used for *in silico* mutagenesis (Nomaguchi et al., 2013b). Changes in the stability **(A)** and affinity **(B)** scores by single amino acid substitutions were computed by using the Protein Design application in MOE as described in section “Materials and Methods.” Bars indicate standard deviations of the score (*n* = 3).

To address the influence on gp120 binding to CD4, we constructed a 3-D model of 562 full-length gp120 bound to cynomolgus monkey CD4 (GenBank accession no. D63349), and performed *in silico* mutagenesis as described in the study of HIV-1 capsid protein (Nomaguchi et al., 2013b). Four point mutations (N132K in V1/F174L in V2/S304G in V3/I307V in V3) were predicted to reduce the stability of gp120-CD4 complex, whereas the other three (L124F in V1/G150R in V1/G310R in V3) were expected to increase the stability (**Figure 8A**). Thus, some adaptive mutations were assumed to be disadvantageous in terms of the stability of CD4 bound structure. Nevertheless, they appeared reproducibly during adaptation in macaque cells (Nomaguchi et al., 2013a), indicating that some advantage(s) surpasses the harmful effect. Affinity scores predicted that the point mutations L124F and G150R in V1, which increase the stability of gp120-CD4 complex (**Figure 8A**), also increase the binding affinity of gp120 and CD4 (**Figure 8B**). These results suggest that these V1 mutations can cause structural changes in gp120 to improve CD4 binding.

**FIGURE 9:**
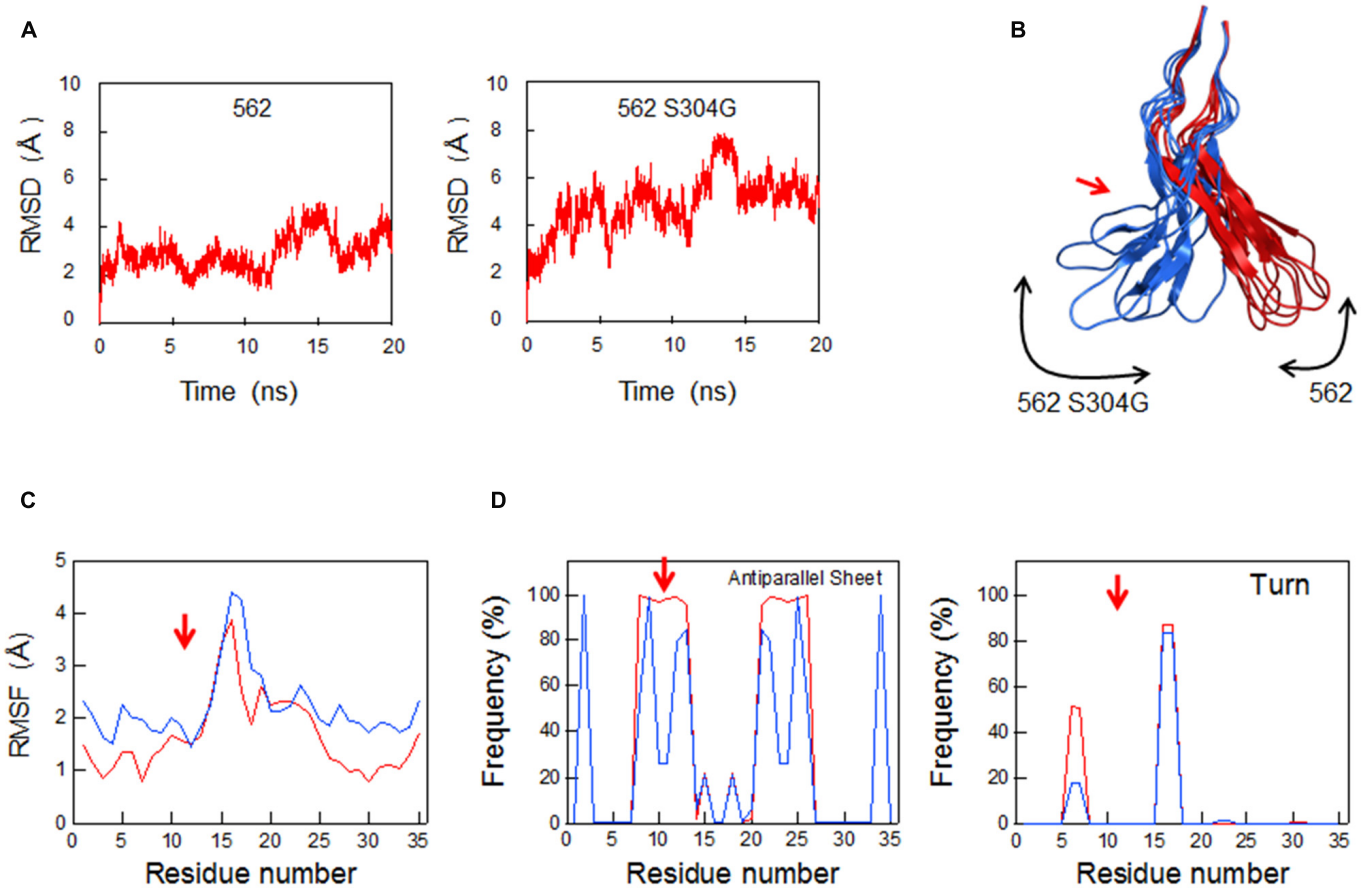
**Effects of S304G mutation on molecular dynamics of V3 loop.** Molecular models for 562 and 562 S304G V3 loops were constructed by homology modeling and subjected to MD simulations as described in section “Materials and Methods.” **(A)** Time course of RMSD between the initial model and models at given times of MD simulation. **(B)** Superposition of V3 structures obtained during 10–20 ns of MD simulations. **(C)** Distribution of RMSF in V3 loop. RMSF values that represent atomic fluctuations of the main chains of individual amino acids were calculated with 10,000 snapshots from 10 to 20 ns of each MD simulation. **(D)** Distributions of frequencies of β-sheet and turn structures in V3 loop that were formed during MD simulations. RMSD, RMSF, and frequencies were calculated using the ptraj module in Amber (Case et al., 2005). Red arrows in **(B), (C)**, and **(D)** show the position of S304G mutation. Red and blue lines in **(C)** and **(D)** represent data on 562 and 562 S304G clones, respectively.

V3 loop conformation is critical for interactions with the N-terminal portion of CCR5 (Huang et al., 2005, 2007), and amino acid substitutions within V3 loop can influence its conformational dynamics, changing the viral co-receptor tropism and neutralization sensitivity (Naganawa et al., 2008; Yokoyama et al., 2012; Kuwata et al., 2013). We therefore examined whether V3 adaptive substitutions affect the conformational dynamics of V3 loop using MD simulation (**Figure 9**). We selected S304G substitution in this study, because the substitution most reproducibly and frequently emerged in our adaptation experiments (Nomaguchi et al., 2013a). Introduction of S304G substitution into the V3 stem of 562 clone resulted in enhancement of the RMSD fluctuations (**Figure 9A**) and in rearrangement of the V3 tip (**Figure 9B**). This substitution exchanges serine with glycine, the smallest amino acid residue, and rationally increased magnitudes of the V3 loop fluctuation (**Figure 9C**), leading to reduced probability to form a stable antiparallel β-sheet that contains 5–6 main-chain hydrogen bonds (**Figure 9D**, left panel) and a turn at base/stem region of V3 during the simulations (**Figure 9D**, right panel). These data suggest that S304G substitution can modulate structural features of V3 loop for co-receptor interactions.

## Discussion

In this study, we combined computational and experimental approaches to address how HIV-1 Env gp120 mechanically modulates viral phenotypes and contributes to viral adaptation. By coupling homology modeling and MD simulation, we constructed full-length gp120 models that disclose key features on the gp120 structure, i.e., topologies of the variable loops (Guttman et al., 2012; Julien et al., 2013; Lyumkis et al., 2013; Pancera et al., 2014; Do Kwon et al., 2015), conformational masking of V3 tip (Guttman et al., 2012; Munro et al., 2014; Pancera et al., 2014), and CD4-induced conformational rearrangement (Huang et al., 2005; Guttman et al., 2012; Julien et al., 2013; Pancera et al., 2014; Do Kwon et al., 2015; **Figures 1–3**). In parallel, cell-based adaptation experiments led to the identification of single amino acid substitutions in Env gp120 for enhanced viral entry/growth through better affinity for CCR5 (**Figures 4–6**). Our structural models predicted that these mutations resided on the receptor binding surfaces and could modulate gp120 structures for receptors binding (**Figures 7–9**). These findings suggest that amino acid substitutions on the receptors binding surface are a key mechanism to tune viral entry efficiency during HIV-1 growth adaptation.

Most importantly, MD simulation of the full-length gp120 molecule revealed a physical function of V1/V2 loop. In the CD4-free state, V1/V2 loop, which initially positioned away from V3 loop, settled into a fixed position near the V3 loop to fit in a thermodynamically stable arrangement (**Figure 1**). The results suggest presence of semi-stable state, i.e., local minimum of energy state for the full-length unliganded gp120. In this semi-stable state, V1/V2 and V3 loops were stably arranged near the core under mutually interactive compact positions (**Figures 1B–D**). This stable arrangement of variable loops on the core is reasonable from the viewpoint of protein chemistry, because unliganded protein generally fold into a compact conformation with minimum protein surface in solution. More importantly, the relative 3-D locations of the variable loops on the core surface in the semi-stable state agree with those reported in the previous experimental studies (Guttman et al., 2012; Julien et al., 2013; Lyumkis et al., 2013; Pancera et al., 2014; Do Kwon et al., 2015) and explain the well-known experimental observation, i.e., conformational masking of V3 neutralization epitope in the trimeric context (Guttman et al., 2012; Munro et al., 2014; Pancera et al., 2014) with keeping CD4 binding site accessible (**Figure 2**). Thus, the present unliganded gp120 model in the semi-stable state recapitulates structural and virological features of the functional gp120 revealed by various experimental methods. Our MD simulation study additionally suggests that V1/V2 loop has an intrinsic physicochemical feature that attracts V3 loop in the CD4-free state. These findings are well consistent with a previous notion that in the CD4-free state, shift to CD4-bound conformation is restrained by interactions of V1/V2 and V3 loops (Kwon et al., 2012). In the CD4-bound state, however, V1/V2 loop repositioned near the bound CD4 to support CD4 binding, while V3 loop formed exposed conformation for co-receptor binding via relief from V1/V2 loop (**Figure 3**). Taken together, these results strongly suggest that V1/V2 loop functions as a nano device to create a gp120 structure that masks co-receptor binding site for immune evasion compatible with maintenance of viral infectivity.

Present study also provides structural bases to understand how V1/V2 and V3 loops mechanically modulate various biological phenotypes of HIV-1. Ligand-free gp120 models (**Figures 1** and **2**) predict that changes in the physicochemical features of V1/V2 or V3 loop, by amino acid substitutions or glycosylation, would alter the nature of attractive interactions among these loops in the CD4-free state. Such changes in turn could cause changes in the exposure levels of co-receptor binding site on virions and thereby would alter viral neutralization sensitivity to anti-V3 antibodies (Etemad-Moghadam et al., 1999; Pinter et al., 2004; Krachmarov et al., 2006; Naganawa et al., 2008), as well as fusogenic activity of gp120 and viral infectivity (Sullivan et al., 1993; Ogert et al., 2001; Laird et al., 2008; Cavrois et al., 2014). In addition, CD4-bound gp120 models (**Figure 3**), *in silico* mutagenesis of gp120 (**Figure 8**), and MD simulation of V3 loop (**Figure 9**) suggest that changes in the loops could alter the efficiency of receptors binding in the CD4-bound state. For example, L124F and G150R substitutions in V1 loop were predicted to increase gp120 binding affinity for CD4 (**Figure 8**), and S304G substitution in V3 loop was expected to alter conformational dynamics of V3 loop (**Figure 9**). Although structural impacts by the adaptive mutations remained characterized further, present findings suggest that a combination(s) of multiple rather than a single mechanism, such as alterations in physical interactions among V1/V2 and V3 loops in the unliganded state and/or alterations in physical interactions with CD4/CCR5, governed the modulation of growth phenotypes of Env-gp120 mutants in this study.

Present study further provides structural insights into the HIV-1 adaptation. Considering that V3 tip constitutes a potent neutralization epitope (Goudsmit et al., 1988; Palker et al., 1988; Rusche et al., 1988; Javaherian et al., 1989), HIV-1 needs to keep evading swarms of newly generated anti-V3-tip antibodies against replicating viruses during persistent infection in infected individuals. A key evasion mechanism is considered to be masking of the co-receptor binding site in V3 (Bou-Habib et al., 1994; Stamatatos and Cheng-Mayer, 1995; Krachmarov et al., 2005; Lusso et al., 2005), i.e., V3 tip. Meanwhile, persisting viruses also should maintain their infectivity that requires the exposure of the co-receptor binding site (Huang et al., 2005), i.e., V3 tip. As suggested in the present study, V1/V2 loop seems to be an essential device to create structural compatibility of gp120 for these reciprocal requirements. Considering that V1/V2 and V3 loops are intrinsically highly variable (HIV Sequence Database^3^), it is conceivable that configurations of V1/V2 and V3 loops would also have variations in nature. The structural variations on the gp120 surface generated by variable loops would in turn allow natural selection of the fittest viruses as suggested in previous (Nomaguchi et al., 2013a) and present (**Figures 5–9**) studies on the HIV-1 Env adaptation. Thus, our structural and virological data are consistent with the assumption that variable V1/V2 and V3 loops are key devices to create structural variations of the gp120 surface to optimize replication fitness under given environments.

The authentic amino acid residues, at the sites that acquired adaptive mutations are located, are relatively well-conserved among virus strains from all HIV-1 subtypes (Zolla-Pazner and Cardozo, 2010; for subtype B, see **Figure 4B**). Interestingly, both F(Phe)/L(Leu) at position 174 and both S(Ser)/G(Gly) at position 304 exist as natural variants (**Figure 4B**). Mutation of F174 residue has been shown to affect binding of a V2-specific antibody 697-D and of neutralizing antibodies PG9 and PG16 (Zolla-Pazner and Cardozo, 2010). It is intriguing to see how the replication of viruses carrying F174L mutation is regulated in individuals. Notably, a particular V3 mutation (S304G) reproducibly appeared with high frequency in the long-term cultures of 562-infected macaque cell lines, and enhanced viral replication ability not only in macaque cells but also in human cells (Nomaguchi et al., 2013a). Even for primary HIV-1 isolates from infected patients, serine and glycine at this position coexist within an individual patient in some cases (Troyer et al., 2005; Delobel et al., 2007; Recordon-Pinson et al., 2013). These results suggest that the S304G mutation is a very powerful mutation to increase replication fitness of R5-tropic HIV-1 virus in primate cells. Nevertheless, this mutation has been reported to be minor in an R5-tropic virus population in humans (serine, 88% and glycine, 12%; Hung et al., 1999) and in SHIV_SF162P3_-infected macaques (Harouse et al., 2001; Ho et al., 2007; Tasca et al., 2008). These findings suggest the presence of some selective pressure(s) that restrict overgrowth of the S304G variant *in vivo*. In this regard, we previously reported that V3 mutation is very potent in modulating fluctuation and conformation of the interaction surface of gp120 outer domain (Naganawa et al., 2008; Yokoyama et al., 2012). Therefore, the S304G mutation may augment efficiency of gp120 binding not only to co-receptors but also to neutralization antibodies. Further study is necessary to address this issue.

In summary, our *in silico* analysis revealed the impact of HIV-1 Env-gp120 MD on viral replication. We show here structural and virological evidences that V1/V2 and V3 loops of HIV-1 Env gp120 function as key nano devices to create the Env structure suitable for immune escape compatible with maintenance of viral infectivity, or to acquire adaptive mutations for enhancing viral replication ability. Interlinking *in silico* science with virology is a powerful strategy for better understanding of HIV-1 adaptation and its biological significance.

## Author Contributions

MY: design of the work; acquisition, analysis, and interpretation of data for the work; drafting the work; final approval of the manuscript; agreement to be accountable for all aspects of the work. MN: design of the work; acquisition, analysis, and interpretation of data for the work; drafting the work; final approval of the manuscript; agreement to be accountable for all aspects of the work. ND: acquisition and analysis of data for the work; revising the work; final approval of the manuscript; agreement to be accountable for all aspects of the work. TK: design of the work; revising the work; final approval of the manuscript; agreement to be accountable for all aspects of the work. AA: design of the work; interpretation of data for the work; drafting the work; final approval of the manuscript; agreement to be accountable for all aspects of the work. HS: design of the work; interpretation of data for the work; drafting the work; final approval of the manuscript; agreement to be accountable for all aspects of the work.

## Conflict of Interest Statement

The authors declare that the research was conducted in the absence of any commercial or financial relationships that could be construed as a potential conflict of interest.

## Footnotes

1 https://www.chemcomp.com/journal/align.htm

2 http://glycam.org/

3 http://www.hiv.lanl.gov/content/sequence/HIV/mainpage.html
